# Analysis of Prognostic Factors of World Health Organization Grade Ⅲ Meningiomas

**DOI:** 10.3389/fonc.2020.593073

**Published:** 2020-12-07

**Authors:** Weidong Tian, Jingdian Liu, Kai Zhao, Junwen Wang, Wei Jiang, Kai Shu, Ting Lei

**Affiliations:** ^1^Department of Neurosurgery, Tongji Hospital, Tongji Medical College, Huazhong University of Science and Technology, Wuhan, China; ^2^Department of Neurosurgery, The First Affiliated Hospital, School of Medicine, Shihezi University, Shihezi, China

**Keywords:** meningioma, malignancy, prognosis, recurrence, WHO

## Abstract

**Objective:**

WHO grade III meningiomas are highly aggressive and lethal. However, there is a paucity of clinical information because of a low incidence rate, and little is known for prognostic factors. The aim of this work is to analyze clinical characteristics and prognosis in patients diagnosed as WHO grade III meningiomas.

**Methods:**

36 patients with WHO grade III meningiomas were enrolled in this study. Data on gender, age, clinical presentation, preoperative Karnofsky Performance Status (KPS), histopathologic features, tumor size, location, radiologic findings, postoperative radiotherapy (RT), surgical treatment, and prognosis were retrospectively analyzed. Progression-free survival (PFS) and overall survival (OS) were evaluated using the Kaplan-Meier method. Univariate and multivariate analysis were conducted by the Cox regression model.

**Results:**

Median PFS is 20 months and median OS is 36 months in 36 patients with WHO grade III meningiomas. Patients with secondary tumors which transformed from low grade meningomas had lower PFS (p=0.0014) compared with primary group. Multivariate analysis revealed that tumors location (PFS, p=0.016; OS, p=0.013), Ki-67 index (PFS, p=0.004; OS, p<0.001) and postoperative radiotherapy (PFS, p=0.006; OS, p<0.001) were associated with prognosis.

**Conclusion:**

WHO grade III meningiomas which progressed from low grade meningiomas were more prone to have recurrences or progression. Tumors location and Ki-67 index can be employed to predict patient outcomes. Adjuvant radiotherapy after surgery can significantly improve patient prognosis.

## Introduction

Meningiomas arise from the arachnoidal cap cells, as one of most common primary tumors of the central nervous system, comprising at least 20% of all brain tumors (1, 2). World Health Organization classification of tumors of the central nervous system has undergone multiple revisions since 2000. According to the newest 2016 WHO classification criteria, meningiomas are divided into three grades and 15 pathological subtypes (2–4). Although most of meningiomas are benign, 1%–3% are malignant (WHO grade III) with high recurrence and mortality rates, and may even spread by metastatic dissemination (1, 5–8). WHO Grade III meningiomas consist of three pathological subtypes, namely, anaplastic, papillary, and rhabdoid, of which anaplastic meningiomas is the most common (2–4).

The prognostic factors and clinical characteristics of WHO grade III meningiomas are still unclear and controversial due to its rarity. In this retrospective study, long-term follow-up data of 36 patients with WHO grade III meningiomas who were diagnosed and treated in our department were obtained and analyzed. As far as we know, this is one of the largest retrospective studies to be conducted and may increase the understanding of prognosis of WHO grade III meningiomas.

## Methods

### Patients and Data Acquisition

From January 2011 to May 2018, 36 patients with WHO grade III meningiomas were operated in the neurosurgery department of Tongji Hospital, Wuhan, China. Patients data including gender, age at time of surgery, clinical presentation, preoperative Karnofsky Performance Status (KPS), histopathologic features, tumor size, location, radiologic findings, postoperative radiotherapy (RT), surgical treatment, and prognosis were retrospectively reviewed. In addition, patients were divided into two groups: a primary group and a secondary group, and the latter has tumors which transformed from grade I or II meningiomas. The extent of resection was determined and graded according to the Simpson grading scale by postoperative imaging examination and surgical records. Gross total resection (GTR) was defined as Simpson grades I-II, and subtotal resection (STR) was equivalent to Simpson grades III-V. Tumor size was estimated as the maximal cross-sectional distance from MRI. Extent of peri-tumoral edema was classified as mild (edema adjacent to the tumor less than 1 cm in margin), moderate (1–3 cm), and severe (>3 cm) according to preoperative MRI (9). Progression-free survival (PFS) was calculated from the date of surgery to the time of tumor recurrence or progression. Progression or recurrence was identified when there was 25% increase in size of the enhancing lesion in follow-up MRI after surgery for patients with STR or appearance of new enhancing lesion for patients for GTR. Overall survival (OS) was defined as the time between the date of the surgery and death. The first MRI follow-up was performed one month after surgery, and then the follow-up interval was extended to 3–6 months gradually. Patients alive are censored at last follow-up. Information was obtained *via* outpatient and telephone follow-up. This study was approved by local ethical authorities of Tongji Hospital, Tongji Medical College, Huazhong University of Science and Technology in accordance with the Helsinki Criteria. Written informed consent was obtained from each individual patient.

### Histopathologic Examination

The diagnosis was confirmed by neuropathology experts through postoperative histopathologic examination of tumor samples according to 2016 WHO grading criteria of CNS tumors. Monoclonal antibodies immunohistochemical staining was used to detect expression of vimentin (VIM), epithelial membrane antigen (EMA), S-100, and Ki-67. Unfortunately, the results of S-100 detection failed to be obtained in 4 patients.

### Statistical Analysis

Possible prognostic factors included age, gender (male or female), group (primary or secondary), location of tumor (convexity/skull base or others), size of tumor (<5 cm or ≥5 cm), pathological subtype (anaplastic or papillary/rhabdoid), bone invasion (yes or no), peri-tumoral edema (none/mild or moderate/severe), extent of surgery (STR or GTR), postoperative radiotherapy (yes or no), preoperative KPS (<80 or ≥80), ki-67 index (≤30% or >30%). Statistical analysis was conducted using Kaplan-Meyer method to estimate the survival curves and log-rank test was used to compare the differences between groups. The effects of each variable on PFS and OS were also analyzed by univariate and multivariate cox regression analysis. The stepwise backward method was used in multivariate regression model, and the variables with a P>0.15 were removed. A p value less than 0.05 was considered significantly different. R software (version 4.0.2) was used to perform all statistical analysis.

## Results

### Patients and Tumor Characteristics

**Figure 1:**
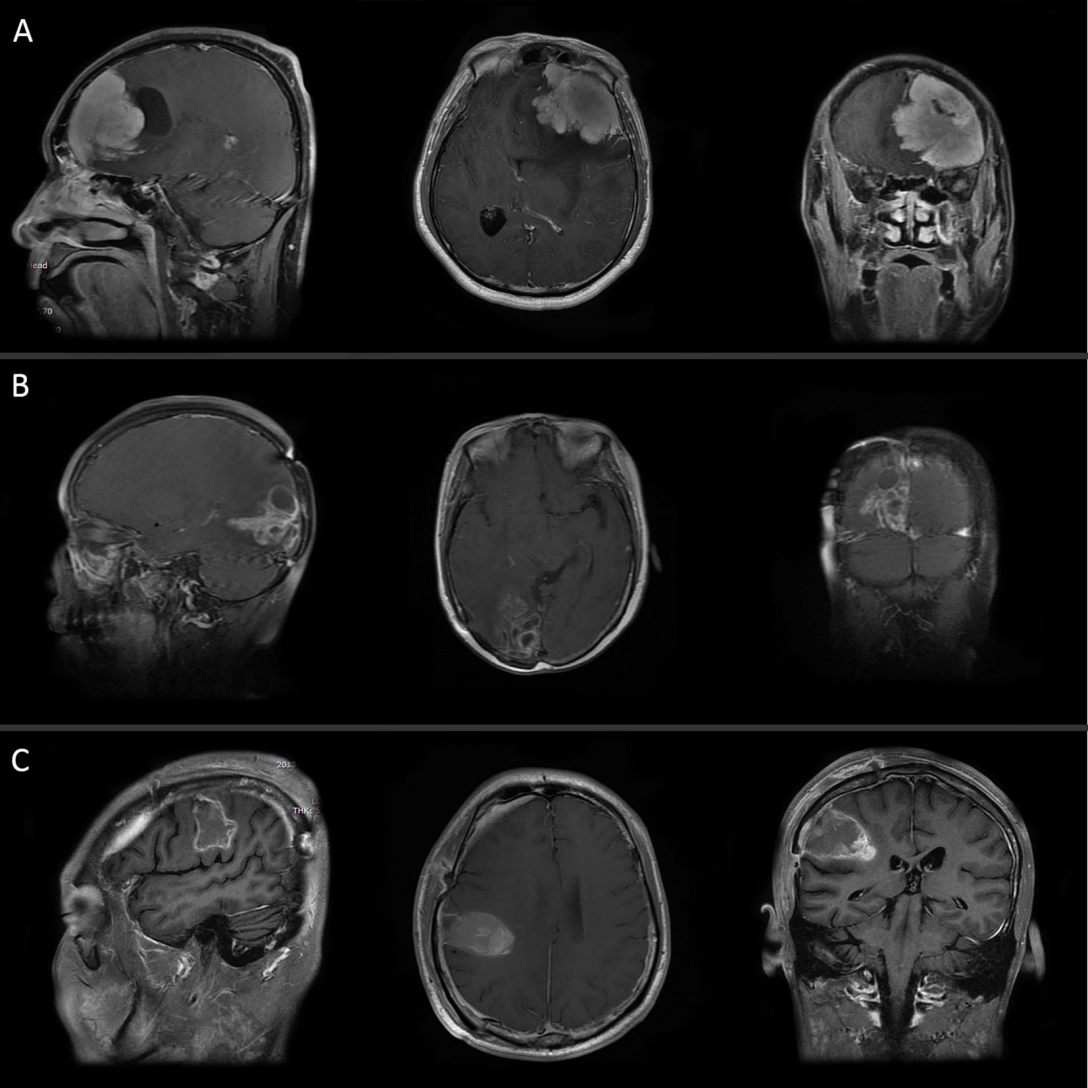
MRI of three pathological subtypes: **(A)** anaplastic meningioma, **(B)** papillary meningioma, and **(C)** rhabdoid meningioma.

The clinical data of 36 patients with WHO grade III meningiomas are shown in **Table 1**. 15 were male and 21 were female with a male to female ratio of 1:1.4, and the median age at surgery was 48.5 years (range, 19–75 years). 5 patients (13.9%) with grade III meningiomas which transformed from low grade meningiomas (grade I or II) were assigned to a secondary group, while the remaining 31 patients (86.1%) were defined as a primary group. Headache was the most common symptom, and other symptoms included limb weakness, epilepsy, visual disturbance, and aphasia. Tumor location was divided into convexity (17 cases, 47.2%), parasagittal/falx (10 cases, 27.8%), skull base (5 cases, 13.9%), and posterior fossa (4 cases, 11.1%). The mean of the maximum of diameter of tumor was 5.11 ± 1.83 cm (range, 3–12 cm). Bone invasion was identified in 6 patients (skull base vs. convexity vs. posterior fossa: 3 vs. 2 vs. 1). Eleven patients displayed mild peri-tumoral edema, while moderate and severe peri-tumoral were observed in 14 and 5 patients, respectively. Brain or bone invasion and peri-tumoral edema were observed on MRI in three pathological subtypes as shown in **Figure 1**. Twenty-four patients (66.7%) were treated with GTR, while STR was achieved in 12 patients. Only 5 patients (all in secondary group) had prior surgical resection of low grade meningioma. Among them, 3 patients were treated with GTR and 2 patients underwent STR. After surgery, 7 patients with STR and 12 patients with GTR underwent fractionated radiotherapy.

**Table 1:** Patients and tumor characteristics of 36 patients with World Health Organization-grade III meningiomas.

Characteristics	Numbers(%)
**Gender**	
Male	15(41.7%)
Female	21(58.3%)
**Group**	
Primary	31(86.1%)
Secondary	5(13.9%)
**Pathological subtype**	
Anaplastic	26(72.2%)
Papillary	5(13.9%)
Rhabdoid	5(13.9%)
**Location of tumor**	
Convexity	17(47.2%)
Parasagittal/falx	10(27.8%)
Skull base	5(13.9%)
Posterior fossa	4(11.1%)
**Size of tumor (maximum diameter of tumor)**	
<5 cm	16(44.4%)
≥5 cm	20(55.6%)
Mean ± SD	5.11 ± 1.83
**Extent of surgery**	
GTR	24(66.7%)
STR	12(33.3%)
**KPS**	
<80	11(30.6%)
≥80	25(69.4%)
**Bone invasion**	
Yes	6(16.7%)
No	30(83.3%)
**Peritumoral edema**	
None	6(16.7%)
Mild	11(30.6%)
Moderate	14(38.9%)
Severe	5(13.9%)
**Postoperative radiotherapy**	
Yes	19(52.8%)
No	17(47.2%)

### Histopathologic Findings

26 anaplastic meningiomas, 5 papillary meningiomas and 5 rhabdoid meningiomas were diagnosed through postoperative histopathologic examination according to 2016 WHO grading criteria of CNS tumors. The levels of expression of VIM, EMA and S-100 was classified by immunohistochemical staining as follows: positive (+), weakly positive (±), and negative (−). All of 36 patients were positive for VIM. EMA was positive in 35 cases (97.2%). The results of S-100 detection were only available in 32 patients, among which 18 patients (56.3%) were weakly positive or positive. The mean Ki-67 index was 30.2% (range, 2%–70%).

### Progression-Free Survival and Overall Survival

The median follow-up period was 26.5 months (range, 12–66 months) among 36 patients with WHO grade III meningiomas. Tumor progression was observed in 27 patients (75.0%) and 20 patients (55.6%) died by the time of last follow-up. The median PFS and OS of 36 patients were 20 months (range, 6–53 months) and 36 months (range, 12–66 months), respectively.

**Figure 2:**
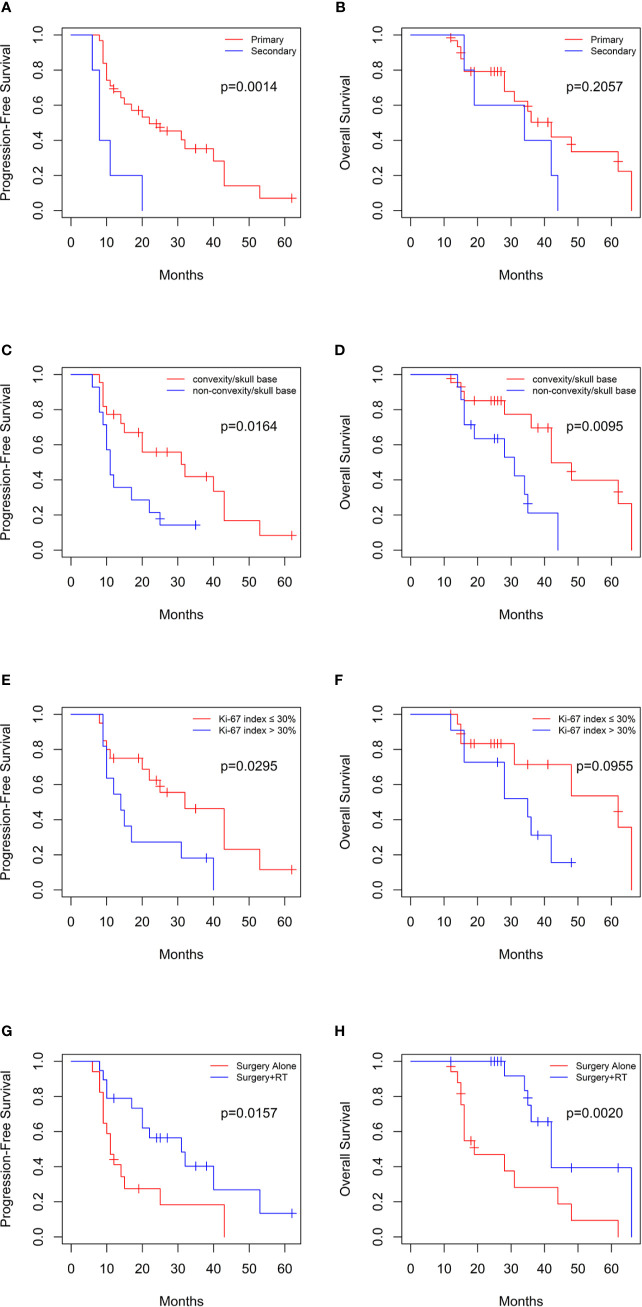
Kaplan-Meier curves for progression-free survival and overall survival based on **(A, B)** primary or secondary group, **(C, D)** tumor location in 36 patients, **(E, F)** Ki-67 index in primary group, and **(G, H)** postoperative radiotherapy.

The Kaplan–Meier survival analysis indicated that the patients with secondary tumors which transformed from low grade meningomas had lower PFS compared with primary group (median PFS: 8 vs. 22 months, log-rank p=0.0014), while the difference in the OS was not significant (median OS: 34 vs. 42 months, log-rank p=0.2057) (**Figures 2A, B**). Moreover, tumors located in the convexity/skull base were associated with better PFS (median PFS: 31 vs. 11 months, log-rank p=0.0164) and OS (median OS: 42 vs. 31 months, log-rank p=0.0095) (**Figures 2C, D**). Although the Ki-67 index were not significantly correlated with PFS and OS, data was significantly different in PFS (median PFS: 32 vs. 14 months, log-rank p=0.0295) but not OS (median OS: 62 vs. 35 months, log-rank p=0.0955) when analysis was conducted in primary group (**Figures 2E, F**). In addition, postoperative radiotherapy significantly prolonged PFS (median PFS: 31 vs. 11 months, log-rank p=0.0157) and OS (median OS: 42 vs. 19 months, log-rank p=0.0020) of patients (**Figures 2G, H**).

Univariate and multivariate cox regression analysis yielded similar results as shown in **Table 2**. On multivariate regression modeling, secondary tumors (hazard ratio[HR]=4.19, p=0.008), parasagittal/falx or posterior fossa meningiomas (HR=3.14, p=0.016), and high Ki-67 index (HR=3.95, p=0.004) remained significantly correlated with risk of progression. On the contrary, postoperative RT (HR=0.28, p=0.006) conferred a protection against tumor progression. In terms of OS, independent risk factors included location (HR=3.69, p=0.013), Ki-67 index (HR=10.08, p<0.001), and postoperative RT (HR=0.09, p<0.001). However, the effects of age, gender, pathological subtype, tumor size, bone invasion, peri-tumoral edema, extent of surgery and preoperative KPS on PFS and OS have not been demonstrated by univariate and multivariate analysis.

**Table 2:** Results of univariate and multivariate Cox regression analysis.

	PFS-Univariate Analysis			PFS-Multivariate Analysis		
Factors	HR	95%CI	P value	HR	95% CI	P value
Age	1.00	0.97–1.03	0.862	–	–	–
Gender (male vs. female)	1.24	0.57–2.72	0.591	–	–	–
Group (primary vs. secondary)	4.76	1.69–13.36	0.003*	4.19	1.45–12.07	0.008*
Location (convexity/skull base vs. others)	2.64	1.15–6.08	0.022*	3.14	1.24-7.92	0.016*
Size (<5 cm vs. ≥5 cm)	1.74	0.78–3.84	0.173	–	–	–
Pathological subtype (anaplastic vs. others)	1.25	0.55–2.83	0.599	–	–	–
Extent of surgery (STR vs. GTR)	0.65	0.29–1.44	0.29	–	–	–
Preoperative KPS (<80 or ≥80)	1.67	0.7–3.97	0.246	–	–	–
Ki-67 index (≤30% or >30%)	2.16	0.96–4.84	0.062	3.95	1.55–10.09	0.004*
Bone invasion	0.61	0.22–1.65	0.328	–	–	–
Peritumoral edema	0.75	0.35–1.62	0.463	–	–	–
Radiotherapy	0.38	0.17–0.85	0.018*	0.28	0.11–0.69	0.006*
	**OS-Multivariate Analysis**			**OS-Multivariate Analysis**		
	**HR**	**95%CI**	**P value**	**HR**	**95% CI**	**P value**
Age	0.99	0.96–1.02	0.553	–	–	–
Gender (male vs. female)	1.54	0.62–3.81	0.35	–	–	–
Group (Primary vs. secondary)	1.94	0.68–5.53	0.214	–	–	–
Location (convexity/skull base vs. others)	3.5	1.27–9.63	0.015*	3.69	1.32–10.35	0.013*
Size (<5 cm vs. ≥5 cm)	1.47	0.57–3.77	0.423	–	–	–
Pathological subtype (anaplastic vs. others)	1.13	0.44–2.91	0.796	–	–	–
Extent of surgery (STR vs. GTR)	0.68	0.27–1.72	0.413	–	–	–
Preoperative KPS (<80 or ≥80)	1.37	0.49–3.82	0.543	–	–	–
Ki-67 index (≤30% or >30%)	2.0	0.77–5.18	0.152	10.08	2.74–37.13	<0.001*
Bone invasion	0.54	0.17–1.70	0.294	–	–	–
Peritumoral edema	1.40	0.56–3.48	0.475	–	–	–
Radiotherapy	0.24	0.09–0.65	0.005*	0.09	0.02–0.31	<0.001*

P* values are statistically significant. HR, hazard ratio; CI, confidence interval.

## Discussion

WHO grade III meningioma is a type of malignant tumors characterized by a high rate of recurrence and lethality regardless of the therapeutic regimen (5). Median PFS and OS were 14.5–46 and 32–59 months respectively in previous literatures (7, 10–12), which is similar to our data. Orton et al. reported that increasing age was significantly associated with lower OS (13). Balasubramanian and his colleagues have shown that a preoperative KPS of <80 was related to poor prognosis (7). However, the effect of age and preoperative KPS on PFS and OS was not observed in our study and other reports (10–12). Although female was considered as one of the risk factors of benign meningiomas, gender differences was not found in malignant meningiomas (14–16). In contrast, a slight female predominance (a male to female ratio of 1:1.4) is observed in our study. Moliterno et al. suggest female may be more likely to harbor *de novo* WHO grade III meningiomas, which provides a possible explanation for the predominance (17). In accordance with other studies, no significant differences were observed among pathological subtypes in either PFS (p=0.599) or OS (p=0.796) in our results (18).

Several WHO grade III meningiomas can progress from benign or atypical meningiomas (WHO grade I/II) (15, 16, 19). Comparison between primary group and secondary group is performed in this study. The patients with secondary tumors tend to have lower PFS, in agreement with previous reports (17, 19). The intriguing result may partly be explained by TERT promoter mutation. Sahm et al. demonstrated TERT promoter mutations significantly reduced PFS in all WHO grades including WHO grade III meningiomas (20). Additionally, much higher mutation rates of TERT promoter is significantly associated with malignant progression of low grade meningiomas (21). Krayenbühl et al. also found that loss of part or monosomy of chromosome 10 and a significant increase of monosomy or a derivative chromosome 1 in combination with monosomy of chromosome 14 were mainly observed in the patients with malignant progression that had a worse prognosis (22). Overall, these findings implicated that malignant meningiomas which progressed from low grade meningiomas may have distinct genetic profiles that are likely to affect prognosis.

GTR has been proved to be a favorable prognostic factor of atypical meningiomas, while it is controversial in WHO grade III meningiomas (7, 8, 10, 23, 24). Our results showed that extent of surgery did not present a significant effect on prognosis. Moreover, a longer OS in patients with STR was described by several studies (7, 25). The results may be attributable to overly aggressive GTR which results in a higher risk of mortality. In contrast, some authors reported that GTR is significantly related to good prognosis (10, 13). Therefore, maximal safe resection in patients with WHO grade III meningiomas should still be advocated according to Sughrue et al (25).

The beneficial effect of adjuvant RT has been proved by most studies and postoperative RT should be recommended in patients with malignant meningiomas irrespective of the extent of surgery (10, 11, 19). The view is also supported by our results. Patients with postoperative RT had a longer PFS and OS in this study and the report of Zhang et al. (10). Although some transient mild side effects were reported by Adeberg S. et al., the authors suggested that RT should still be offered after initial surgical treatment (26).

As mentioned above in the results, PFS and OS were prolonged in patients with convexity/skull base meningiomas in our study, consistent with the report of Wang et al (27). Parasagittal/falx and posterior fossa meningiomas often were attached to or invade the superior sagittal sinus or transverse sinus and are difficult to resect completely, which leads to poor prognosis (27).

Ki-67 is a proliferation marker, expressed in G1, S, G2, and M phases of cell cycle except G0 phase (28). A high Ki-67 index was recognized as a sign of an adverse prognostic factor in meningiomas. However, the effect of ki-67 index on PFS and OS in WHO grade III meningiomas was not obtained because it was analyzed for benign, atypical, and malignant meningiomas together in some prior studies (27, 29, 30). Our analysis identified that higher Ki-67 index (>30%) is related with increased risk of tumor progression (p=0.003) and death (p=0.008) in patients with WHO grade III meningiomas.

## Conclusion

This study suggested that patients with WHO grade III meningiomas which progressed from low grade tumors have lower PFS. Tumors located in parasagittal/falx and posterior fossa and a high Ki-67 index were correlated to poor prognosis. These factors can be used to predict patient outcomes. In addition, adjuvant radiotherapy after resection prolonged PFS and OS of patients.

## Data Availability Statement

The raw data supporting the conclusions of this article will be made available by the authors, without undue reservation.

## Ethics Statement

The studies involving human participants were reviewed and approved by ethics committee of Tongji Hospital, Tongji Medical College. The patients/participants provided their written informed consent to participate in this study. No animal studies are presented in this manuscript.

## Author Contributions

KS and TL designed and conducted this project. WT, JDL, KZ, JW, and WJ collected the data and run statistical analysis. WT wrote the original manuscript. All authors contributed to the article and approved the submitted version.

## Funding

This work was supported by the National Natural Science Foundation of China (grant nos. 81702478 and 81602204).

## Conflict of Interest

The authors declare that the research was conducted in the absence of any commercial or financial relationships that could be construed as a potential conflict of interest.
